# miR-143-3p targeting of *ITGA6* suppresses tumour growth and angiogenesis by downregulating *PLGF* expression via the *PI3K*/*AKT* pathway in gallbladder carcinoma

**DOI:** 10.1038/s41419-017-0258-2

**Published:** 2018-02-07

**Authors:** Yun-Peng Jin, Yun-Ping Hu, Xiang-Song Wu, Yao-Shi Wu, Yuan-Yuan Ye, Huai-Feng Li, Yong-Chen Liu, Lin Jiang, Fa-Tao Liu, Yi-Jian Zhang, Ya-Juan Hao, Xi-Yong Liu, Ying-Bin Liu

**Affiliations:** 1 Department of General Surgery and Laboratory of General Surgery, Xinhua Hospital, Shanghai Jiao Tong University School of Medicine, No. 1665 Kongjiang Road, 200092 Shanghai, China; 2 Institute of Biliary Tract Disease, Shanghai Jiao Tong University School of Medicine, No. 1665 Kongjiang Road, 200092 Shanghai, China; 3 Department of Gastroenterology, Second Affiliated Hospital of Nanchang University, Nanchang, China; 4 Department of Molecular Pharmacology, City of Hope Comprehensive Cancer Center and Beckman Research Institute, Duarte, CA USA

## Abstract

Gallbladder cancer (GBC) is the most common malignant tumour of the biliary track system. Angiogenesis plays a pivotal role in the development and progression of malignant tumours. miR-143-3p acts as a tumour suppressor in various cancers. Their role in GBC is however less well defined. Here we show that the expression levels of miR-143-3p were decreased in human GBC tissues compared with the non-tumour adjacent tissue (NAT) counterparts and were closely associated with overall survival. We discovered that miR-143-3p was a novel inhibitor of tumour growth and angiogenesis in vivo and in vitro. Our antibody array, ELISA and *PLGF* rescue analyses indicated that *PLGF* played an essential role in the antiangiogenic effect of miR-143-3p. Furthermore, we used miRNA target-prediction software and dual-luciferase assays to confirm that integrin α6 (*ITGA6*) acted as a direct target of miR-143-3p. Our ELISA and western blot analyses confirmed that the expression of *PLGF* was decreased via the *ITGA6/PI3K/AKT* pathway. In conclusion, miR-143-3p suppresses tumour angiogenesis and growth of GBC through the *ITGA6/PI3K/AKT/PLGF* pathways and may be a novel molecular therapeutic target for GBC.

## Introduction

Gallbladder cancer (GBC) is the most common malignant tumour of the biliary track system^1^ and the fifth most common gastrointestinal cancer^2–4^. The Surveillance, Epidemiology, and End Results (SEER) programme has shown the incidence of gallbladder carcinoma to be approximately 2.5 cases per 1×10^5^ people^1–4^. Although the incidence of GBC is lower than the incidences of other gastrointestinal cancers, such as gastric cancer, the survival rate for GBC is poor due to difficulties with early diagnosis, the frequent occurrence of early metastasis and the high degree of malignancy; the five-year survival rate of GBC is less than 5%^5^. Surgical resection is the only effective treatment method because GBC is not sensitive to radiotherapy and chemotherapy^6^. Despite its atypical early symptoms, patients have no opportunity to undergo surgery. Therefore, novel prognostic biomarkers and targeted therapeutics for GBC are necessary^7^.

Angiogenesis plays a central role in the development and progression of malignant tumours^8^. Increases in angiogenic factors and reductions in antiangiogenic factors contribute to the formation of new blood vessels^9^. Vascular endothelial growth factor (*VEGF*) is widely recognized as a significant angiogenic factor. In addition to *VEGF*, another group of proteins, including fibroblast growth factor 2 (*FGF2*), angiopoietin-1 (*Ang-1*) and platelet-derived growth factor receptor-alpha (*PDGFRα*), directly participate in the formation of blood capillaries and lymphatic vessels. The *VEGF* family comprises six secretory glycoproteins, namely, *VEGFA, VEGFB, VEGFC, VEGFD, VEGFE* and placental growth factor (*PLGF*)^10^. Previous studies have reported that *PLGF* plays pivotal roles in pathological contexts such as cancer^11^, whereas *PLGF* actions are redundant in normal physiological processes. *PLGF* binds to and activates VEGF receptor 1 (*Flt-1*) and synergizes the effect of *VEGF*^11^. Numerous small-molecule inhibitors of *VEGF* and *VEGFR2*, including cediranib^12^, sunitinib^13^ and vandetanib^14^, have been approved for therapy. However, significant increases in other angiogenic regulatory factors during cancer development have also been observed following treatment. Thus, a better understanding of the process of angiogenesis is critical to overcoming the side-effects of antiangiogenic approaches and developing a new effective antiangiogenic therapy.

MicroRNAs (miRNAs) are a class of highly conserved small (18–24 nucleotides in length) noncoding RNAs that can inhibit translation or induce mRNA degradation by binding to the 3′-untranslated regions (3’-UTRs) of target genes^15^. As post-transcriptional regulators, miRNAs play significant roles in physiological and pathological processes, including cell differentiation, organ development, cell proliferation, apoptosis and tumourigenesis^16^. miRNAs can act as either oncogenes or tumour suppressors by directly or indirectly modulating cancer genes^17^. Accumulative studies have demonstrated that miRNAs play significant roles in GBC. According to a previous study, miR-143-3p is dramatically downregulated in GBC tissues compared with non-tumour adjacent tissues (NATs)^1,18,19^. However, the precise molecular mechanism through which miR-143-3p influences the progression of GBC remains unknown.

In this study, we aimed to evaluate the underlying roles and mechanisms of miR-143-3p in gallbladder tumourigenesis. We found that the expression level of miR-143-3p was significantly lower in GBC tissues than in the NAT counterparts. Additionally, we found that miR-143-3p played vital roles in GBC angiogenesis and growth. miR-143-3p inhibited the function of integrin α6 (*ITGA6*), a direct target of miR-143-3p, which induced tumour angiogenesis through the *MAPK* and *PI3K/AKT* pathways and enhanced the expression of *PLGF*. Here, we first determined the inhibitory role of miR-143-3p in GBC growth and angiogenesis and then demonstrated the potential use of miR-143-3p as a targeted therapy and as a prognostic indicator for patients with GBC.

## Results

### Downregulation of miR-143-3p is associated with an unfavourable prognosis in GBC patients

**Fig. 1:**
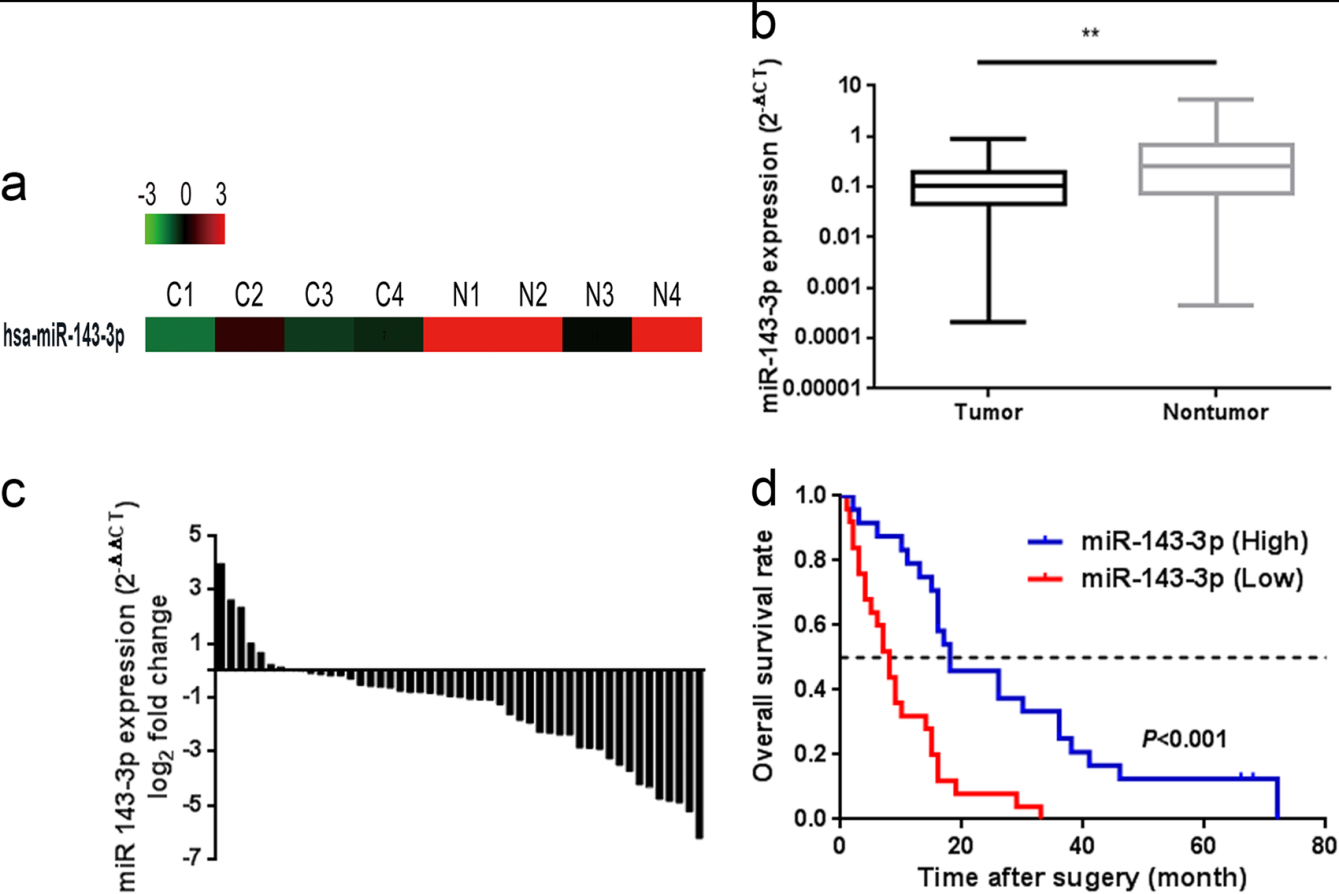
Downregulation of miR-143-3p is correlated with poor clinical outcomes in GBC patients. **a** A portion of the cluster analysis of the miRNA expression profiles of the GBC tissues and NAT counterparts from our previous microarray results. **b** Scatterplots of the relative expression levels of miR-143-3p in the GBC tissues and their corresponding NATs. miR-143-3p expression was calculated and expressed as the miR-143-3p/U6 expression ratio (2^−ΔCT^). *P* = 0.0021. **c** Comparison of the miR-143-3p expression levels between the GBC tissues and their corresponding NATs. **d** Kaplan−Meier overall survival curve of GBC patients based on miR-143-3p expression. *P* < 0.001

According to our previous microarray results^1^, miR-143-3p was dramatically downregulated in GBC tissues compared with NATs (Fig. 1a). To validate our previous miRNA profiling data, we evaluated miR-143-3p expression in 49 paired clinical GBC specimens. The expression levels of miR-143-3p were significantly lower in the tumour tissues than in the corresponding adjacent noncancerous tissues (*P* = 0.0021, Fig. 1b, c). In the survival analysis, 49 GBC patients were divided into two groups (miR-143-3p-low, *n* = 25; miR-143-3p-high, *n* = 24) by setting the cut-off value to the median miR-143-3p expression level. The Kaplan−Meier analysis revealed that high miR-143-3p expression was associated with better overall survival compared with lower expression levels (*P* < 0.001) (Fig. 1d). The mean survival time in the low miR-143-3p expression group was 9.86 months, whereas the mean survival time in the high miR-143-3p expression group was 26.92 months. A clinicopathological association study of the 49 GBCs showed that miR-143-3p was significantly associated with tumour size (*P* = 0.016) (Table 1) and tumour invasion (*P* = 0.023) (Table 1).

**Table 1:** Association of miR-143-3p expression with the clinicopathological features of the GBC patients

Variable	Category	Relative miR-143-3p expression		*χ*2	*P*
		Low (*n* = 25)	High (*n* = 24)		
Age	<60	11	8	0.587	0.561
	≥60	14	16		
Gender	Male	9	10	0.684	0.773
	Female	16	14		
**Tumour size (cm)**	<**3**	**4**	**12**	**6.437**	0.016*
	≥**3**	**21**	**12**		
Histological differentiation	Well	3	6	1.380	0.289
	Moderate or poor	22	18		
**Tumour invasion (AJCC)**	**Tis-T**_**2**_	**8**	**16**	**5.889**	0.023*
	**T**_**3**_ **-T**_**4**_	**17**	**8**		
Lymph node metastasis (AJCC)	Present	9	11	0.490	0.567
	Absent	16	13		

Bold values indicate statistical significance, *P* < 0.05

### miR-143-3p inhibits GBC cell proliferation and angiogenesis in vitro

**Fig. 2:**
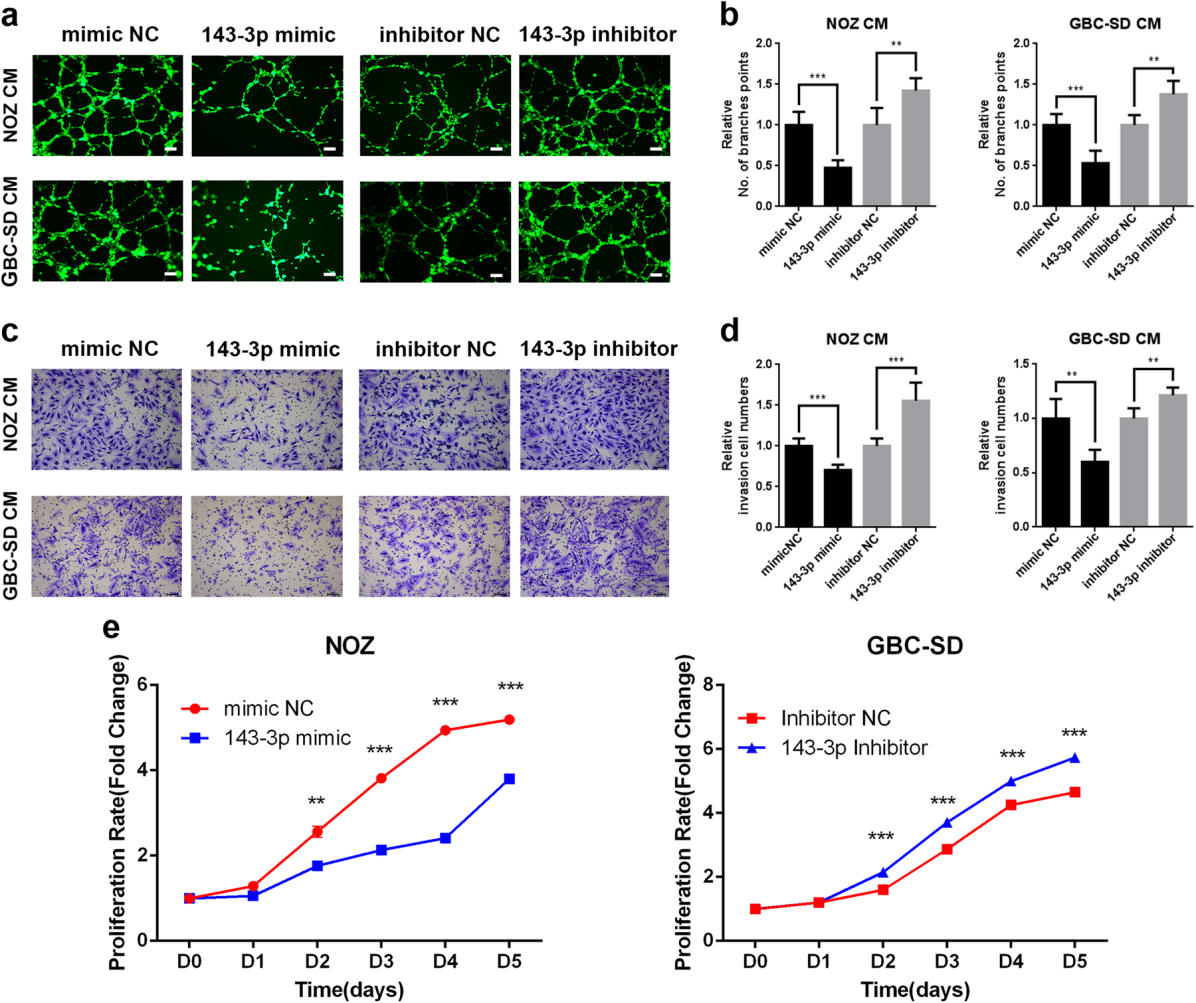
miR-143-3p inhibits GBC cell proliferation and angiogenesis in vitro. **a**, **b** Endothelial tube formation was estimated following incubation of HMVECs with conditioned medium from GBC cells transfected with mimics or inhibitors. The number of branches was quantified (**P < 0.01, ***P < 0.001; Student’s *t* test). Scale bar, 100 µm. **c**, **d** Invasion of HMVECs through the Matrigel chambers after incubation with conditioned medium from miR-143-3p-overexpressing or miR-143-3p-inhibited GBC cells for 48 h. Scale bar, 100 µm. The number of invading cells was calculated and is depicted in the bar graph (**P < 0.01, ***P < 0.001; Student’s *t* test). **e** Cell growth rates over 5 days were determined with CCK-8 proliferation assays (**P < 0.01, ***P < 0.001)

We first assessed the expression levels of miR-143-3p in five human GBC cell lines (NOZ, GBC-SD, SGC996, OCUG-1 and EHGB-1). The miR-143-3p expression levels were lowest in NOZ cells and highest in GBC-SD cells among five GBC cell lines (Supplementary Figure S1a). Therefore, we selected NOZ and GBC-SD cells for subsequent studies. To investigate the biological functions of miR-143-3p in GBC angiogenesis, we performed a gain- and loss-of-function analysis using miR-143-3p mimics and inhibitors. The effects of the mimics and inhibitors were examined using qRT-PCR (Supplementary Figure S1b and c). The conditional medium after 48 h of culture was collected and used for human microvascular vein endothelial cell (HMVEC) tube formation and invasion assays to determine the effects of miR-143-3p on angiogenesis in vitro. As a result, the culture medium from the NOZ and GBC-SD cells transfected with the miR-143-3p mimics significantly impaired capillary tube formation and decreased the invasive abilities of HMVECs (Fig. 2a−d left). Conversely, HMVECs formed more branch points in the group of GBC cells transfected with the miR-143-3p inhibitors than in the inhibitor negative control (NC) group. Additionally, higher invasive activity was evident in HMVECs treated with the conditional medium of GBC cells transfected with the miR-143-3p inhibitors (Fig. 2a−d right). CCK-8 assays were performed to assess the role of miR-143-3p in NOZ and GBC-SD cell proliferation. The GBC cells transfected with the miR-143-3p mimics clearly grew more slowly than the mimic NC group. However, the proliferation rate of GBC cells transfected with the miR-143-3p inhibitors was significantly increased relative to the inhibitor NC group (Fig. 2e and Supplementary Figure 1d). These findings suggest that miR-143-3p suppresses GBC cell proliferation and angiogenesis in vitro.

### miR-143-3p inhibits GBC cell proliferation and angiogenesis in vivo

**Fig. 3:**
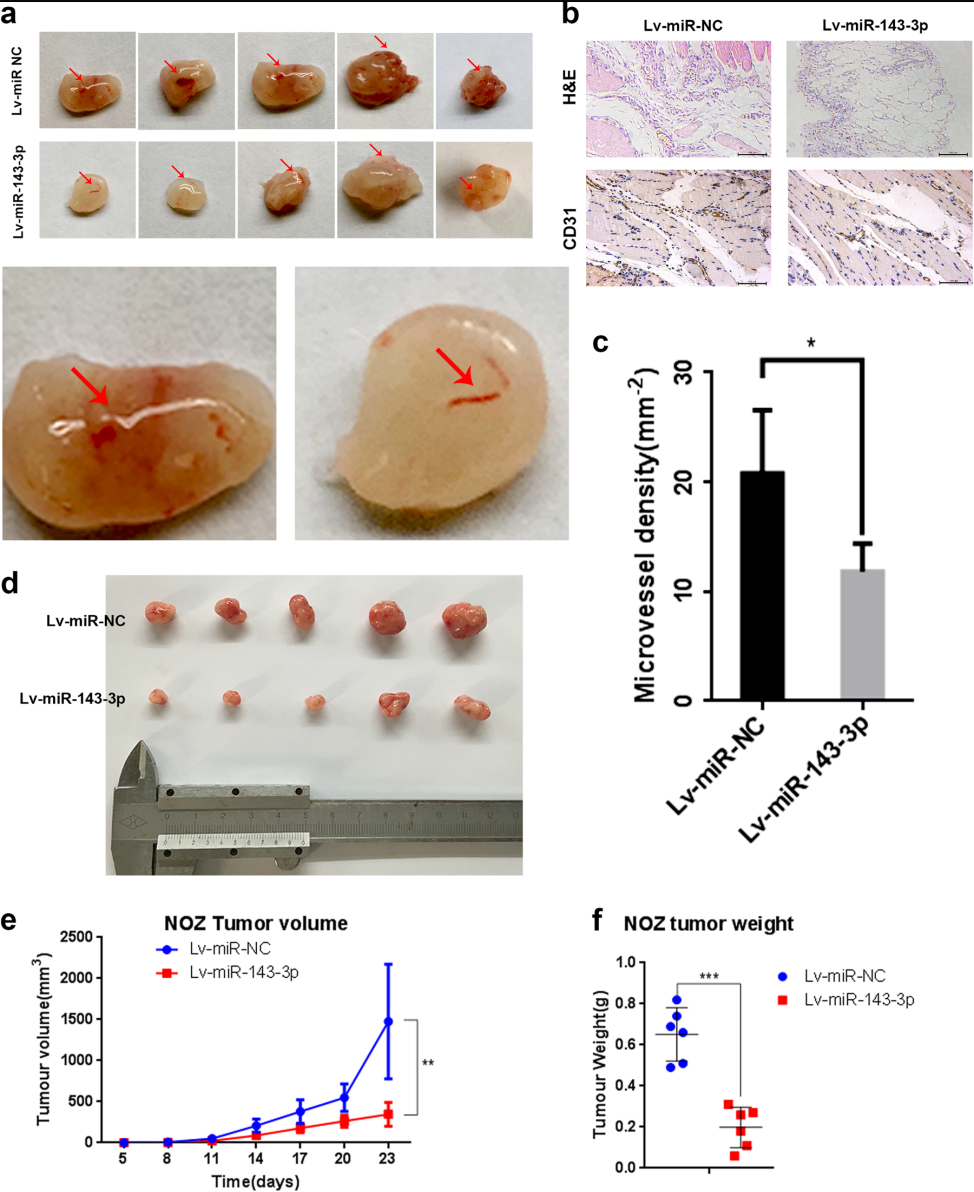
miR-143-3p inhibits GBC cell angiogenesis and proliferation in vivo. **a** Matrigel containing 20 U of heparin and NOZ cells transfected with Lv-miR-NC or Lv-miR-143-3p was subcutaneously implanted in 4−6-week-old male BALB/c athymic nude mice. After 7 days, the Matrigel plugs were removed and photographed. *n* = 5 per group. **b** H&E and *CD31* staining of the Matrigel plug. Scale bar, 100 mm. **c** Quantification of the microvessel density (mm^−2^; *n* = 5; *P < 0.05; Student’s *t* test). **d** Representative examples of tumours formed in nude mice implanted with the indicated cells. **e**, **f** The tumour growth curves are summarized in the line chart. A statistical plot of the average tumour weights in the subcutaneous xenograft model (**P < 0.01, ***P < 0.001, *n* = 5)

To confirm these findings in vivo, NOZ cells stably expressing miR-143-3p were subcutaneously implanted into 4-week-old nude mice to investigate the effect of miR-143-3p on tumour growth. The results showed that the volumes and weights of xenografted tumours were smaller and lower, respectively, in the miR-143-3p overexpression group (Lv-miR-143-3p) than in the NC group (Lv-miR-NC) (Fig. 3d−f). Additionally, a Matrigel plug assay was performed to assess the effects of miR-143-3p on angiogenesis by subcutaneously injecting the mixture of Matrigel and stably expressing miR-143-3p NOZ cells into BALB/c nude mice; immunohistochemistry (IHC) using the *CD31* antibody was performed to evaluate angiogenesis. The result showed that fewer vessels were formed in the miR-143-3p overexpression group (Lv-miR-143-3p) than in the NC group (Lv-miR-NC) (Fig. 3a). Furthermore, immunostaining of the *CD31* protein in the Matrigel plugs of the Lv-miR-143-3p group was remarkably weaker than in the Lv-miR-NC group (Fig. 3b). The vessel densities were lower in the Lv-miR-143-3p plugs than in the Lv-miR-NC plugs (Fig. 3c).

### miR-143-3p inhibits *PLGF*-induced angiogenesis

**Fig. 4:**
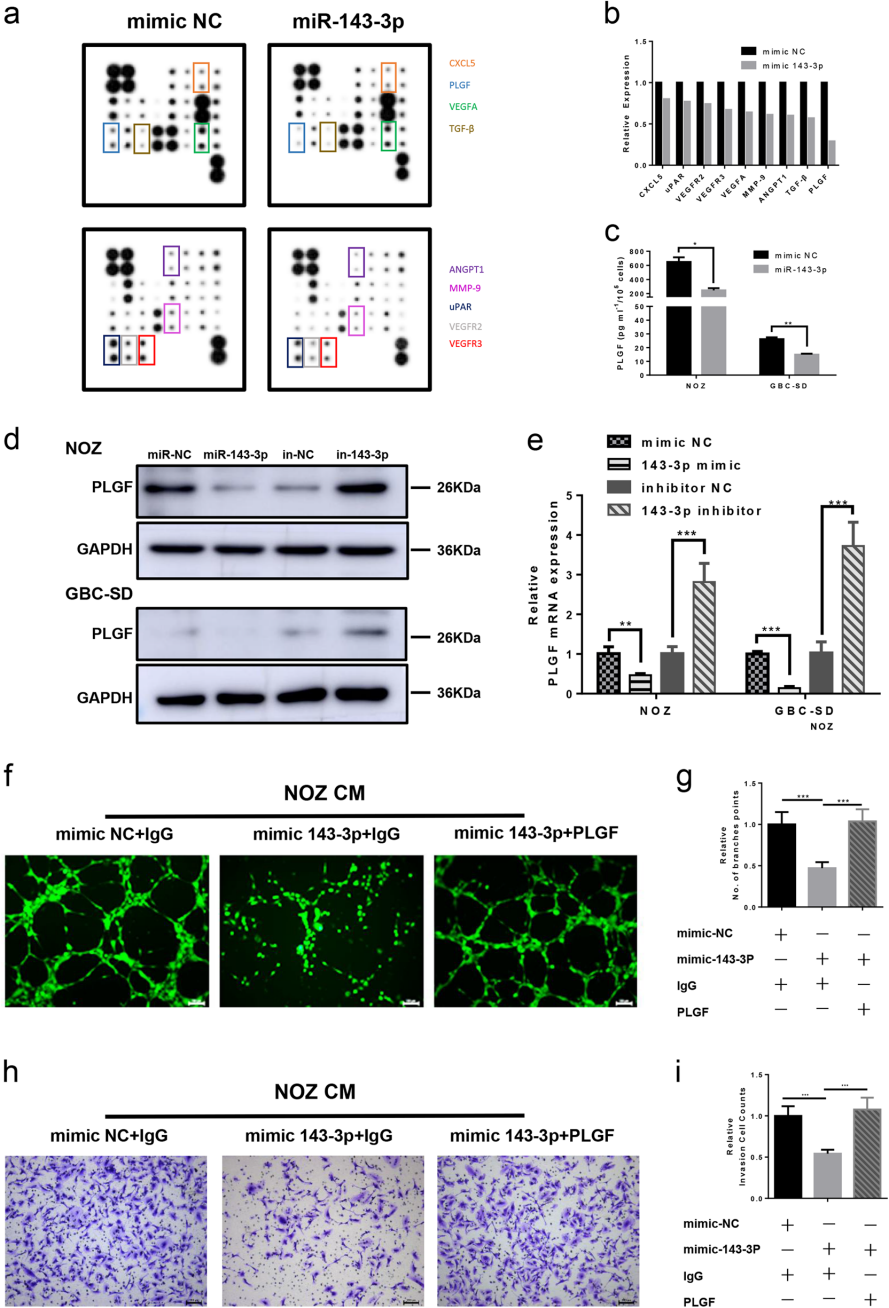
miR-143-3p inhibits expression of *PLGF*. **a** Human angiogenesis array analysis of the conditional medium from NOZ-mimic-NC and NOZ-miR-143-3p mimic cells. **b** A summary of the relative expression levels of the angiogenesis cytokines is provided in the bar graph. **c** *PLGF* in the supernatants of the NOZ and GBC-SD cells that were transfected with mimic NC or the miR-143-3p mimics were quantified by ELISA (*n* = 3; *P < 0.05, **P < 0.01; Student’s *t* test). **d**, **e** *PLGF* expression in the mimic (inhibitor) NC and miR-143-3p mimics (inhibitors)-transfected GBC cells was analysed by western blot and qRT-PCR analysis. *GAPDH* was used as the loading control. **f**, **g** Endothelial tube formation estimation after incubation of HMVECs with conditioned medium from mimic NC or miR-143-3p cancer cells with or without *PLGF*. The number of branches was quantified (*P* < 0.001; Student’s *t* test). **h**, **i** Invasion of HMVECs through the Matrigel chambers after incubation with conditioned medium from mimic NC or miR-143-3p cancer cells with or without *PLGF*. Scale bar, 100 µm. The number of invading cells was determined and is depicted in the bar graph (***P < 0.001; Student’s *t* test)

To determine the role of miR-143-3p in angiogenesis, an angiogenesis antibody array was performed to assess differences in expression of angiogenesis-related cytokines between the conditional medium of NOZ cells transfected with either the mimic NC or miR-143-3p mimics. As shown in Fig. 4a, b, the levels of nine cytokines (*CXCL5*, *uPAR*, *VEGFR2*, *VEGFR3*, *VEGFA*, *MMP-9*, *ANGPT1*, *TGF-β* and *PLGF*) were lower in the NOZ miR-143-3p group compared with the NOZ mimic-NC group. Among them, *PLGF* was dramatically downregulated by approximately 71%. We measured the *PLGF* levels in the cell supernatants after transfection with the miR-143-3p mimics or mimic NC by enzyme-linked immunosorbent assay (ELISA). Consistent with the angiogenesis antibody array data, the *PLGF* level was significantly downregulated in the miR-143-3p mimics group compared with the mimic NC group in both NOZ and GBC-SD cells (Fig. 4c). The *PLGF* mRNA and protein expression levels were determined by qRT- PCR and western blot, and the results indicated that miR-143-3p inhibited the expression of *PLGF* at the transcriptional level (Fig. 4d, e), while silencing of miR-143-3p enhanced the expression of *PLGF* (Fig. 4d, e). Because miR-143-3p inhibits tube formation and invasion, we assessed whether inhibition of angiogenesis occurred via downregulation of *PLGF*. Hence, the recombinant PLGF protein was used to increase *PLGF* expression. The results showed that the addition of recombinant *PLGF* protein significantly increased HMVEC tube formation and invasion, whereas no significant differences were observed between the mimic NC+IgG and miR-143-3p+*PLGF* groups (Fig. 4f−i). No binding sites were found in the 3′UTR of *PLGF*, indicating that *PLGF* was not a direct target of miR-143-3p.

### *ITGA6* is a direct target gene of miR-143-3p

**Fig. 5:**
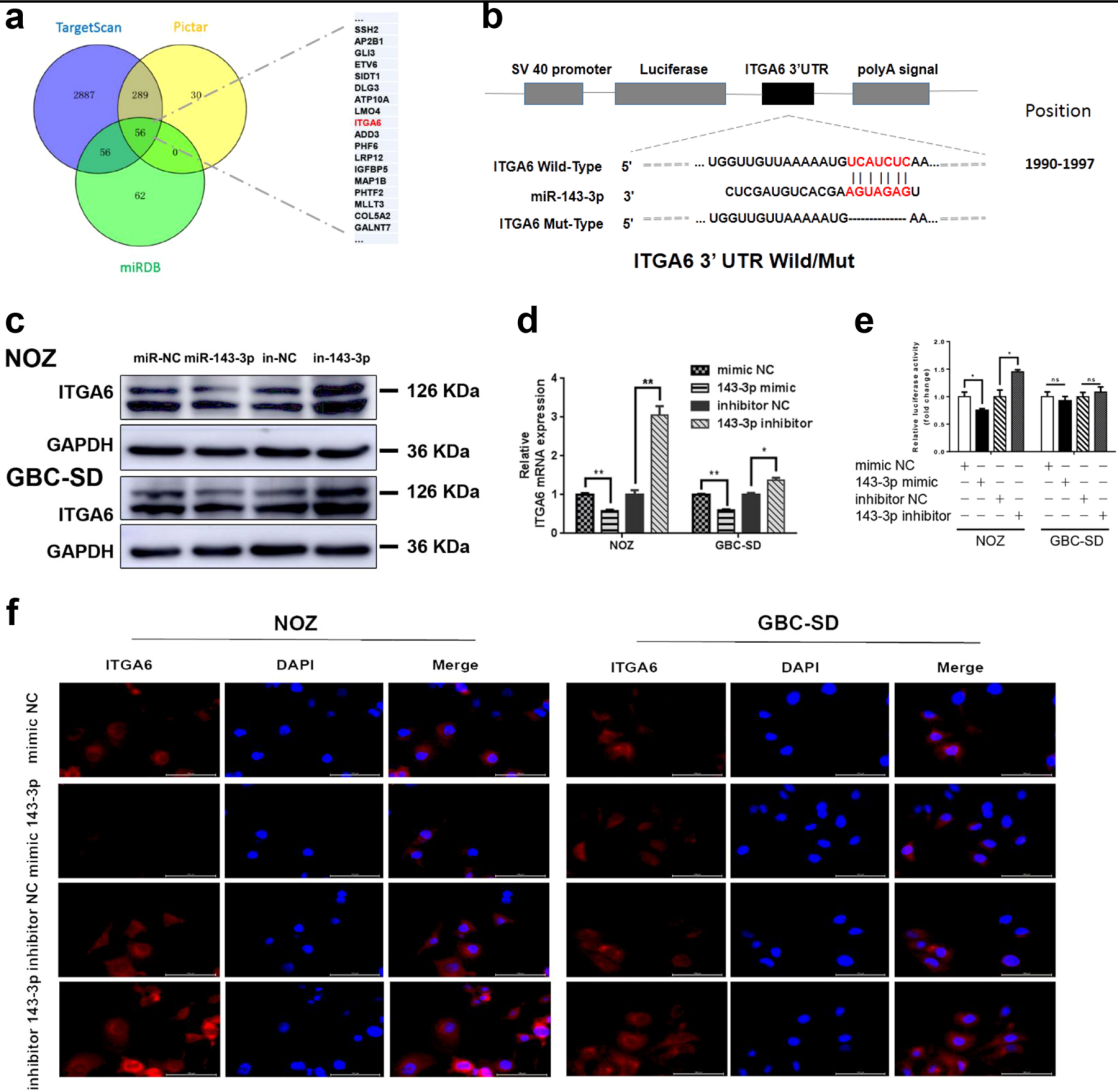
*ITGA6* is a direct target gene of miR-143-3p. **a** Potential miR-143-3p targets predicted by the three miRNA target-prediction programmes (TargetScan, PicTar and miRDB). **b** The wild-type or mutant *ITGA6* 3′UTRs to determine the miR-143-3p binding site. **c**, **d** Overexpression of miR-143-3p attenuates *ITGA6* protein and mRNA expression in NOZ and GBC-SD cells compared with the miRNA-NC group. Silencing of miR-143-3p promotes *ITGA6* protein and mRNA expression in NOZ and GBC-SD cells compared with the miRNA-NC group. (*P < 0.05, **P < 0.01; Student’s *t* test). **e** The relative luciferase activity of the wild-type or mutant *ITGA6* 3′UTR in 293T cells after transfections with the miR-143-3p mimic or inhibitor and corresponding control (*P < 0.05, NS no significant). **f** Expression of *ITGA6* in GBC cells infected with the miR-143-3p mimic or inhibitor and corresponding control was examined by immunofluorescence. The red signals represent *ITGA6* staining. The nuclei were counterstained with DAPI

To explore the molecular mechanisms by which miR-143-3p regulates GBC cell growth and angiogenesis, three miRNA target-prediction programmes (TargetScan, PicTar and miRDB) were used to predict the target sites of miR-143-3p (Fig. 5a). *ITGA6* was among the predicted targets. *ITGA6* is a major component of the ECM signalling pathway, which reportedly promotes tumour angiogenesis^20^. The 1990−1997 region of the *ITGA6* 3′-UTR contains a conserved miR-143-3p binding site (Fig. 5b). Thus, *ITGA6* was selected as a candidate target for further analyses. qRT- PCR and western blot analyses confirmed that miR-143-3p suppressed the endogenous expression of *ITGA6* at the mRNA and protein levels (Fig. 5c, d). Additionally, immunofluorescence assays showed that the expression levels of *ITGA6* in the miR-143-3p mimic-transfected cells were weaker than in the miR-NC-transfected cells. Inhibition of miR-143-3p reversed this phenotype (Fig. 5f). Additionally, the *ITGA6* assessment of the xenografted tumours showed comparable results by IHC (Supplementary Figure S2a). To further confirm that miR-143-3p interacted with the 3′-UTR regions of the *ITGA6* mRNA at the predicted seed sequence binding sites, we constructed a dual-luciferase reporter plasmid containing a fragment of the *ITGA6* 3′-UTR across the conserved miR-143-3p binding sites (Fig. 5b). The dual-luciferase activity assays showed that expression of the *ITGA6* reporter was significantly reduced by the miR-143-3p mimics and increased by the miR-143-3p inhibitors. Conversely, expression of the *ITGA6* reporter containing the mutated sequence of the same fragment was not affected by the miR-143-3p mimics or inhibitors (Fig. 5e). These results indicate that *ITGA6* is a direct downstream target of miR-143-3p.

### *ITGA6* expression is upregulated and negatively associated with miR-143-3p expression in GBC

**Fig. 6:**
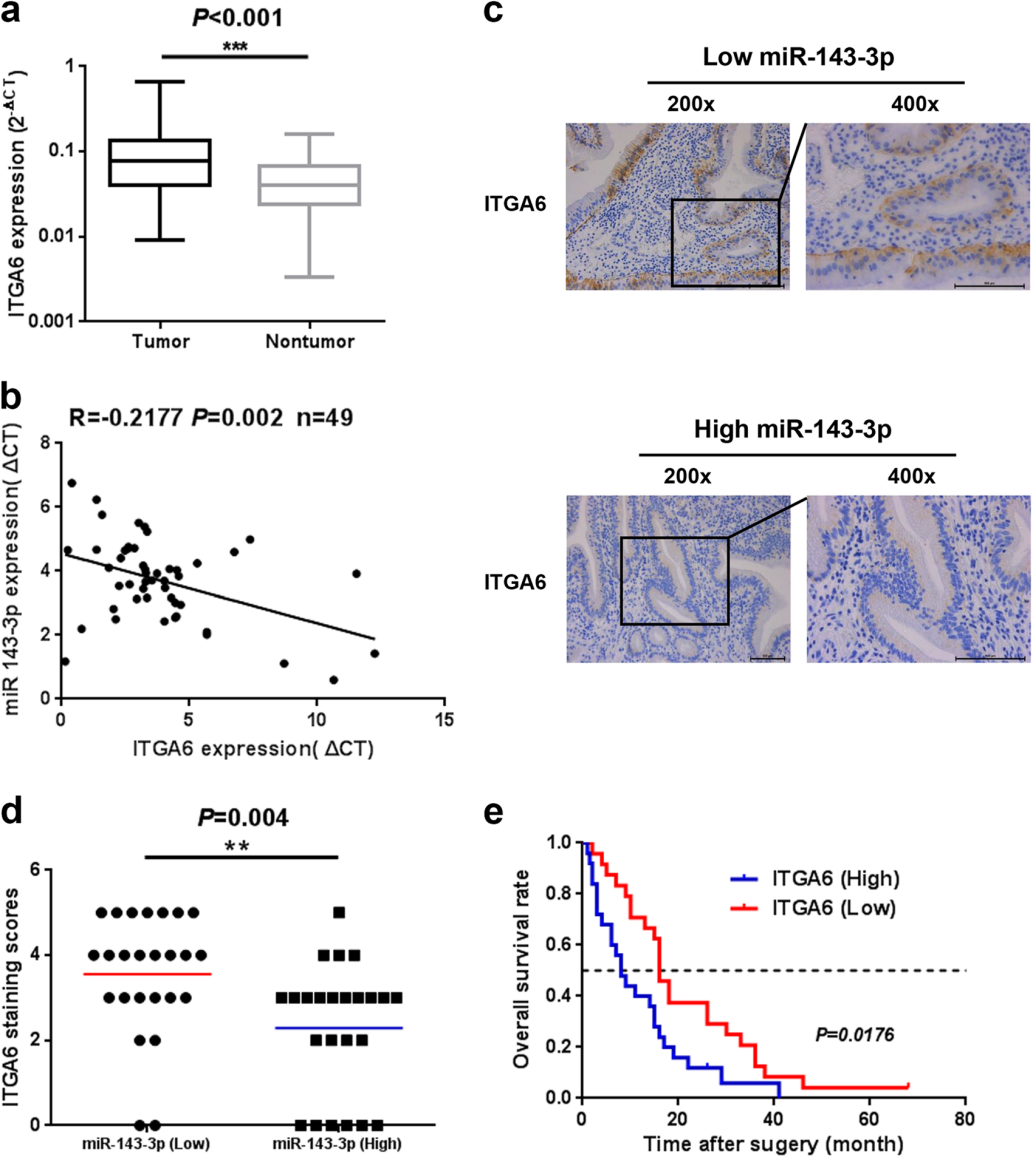
*ITGA6* expression at the mRNA level in human GBC tissues. **a** The *ITGA6* mRNA levels in 49 pairs of GBC tissues and their corresponding NATs. **b** The correlation between the expression levels of miR-143-3p and *ITGA6* was determined using a linear regression analysis and a paired *t* test with the same samples used (*P* < 0.001, *r* = −0.2177, *n* = 49; Pearson’s correlation). **c** Representative IHC micrographs showing *ITGA6* protein expression in GBC tissues with high or low miR-143-3p expression. Scale bar, 100 µm. **d** Scatterplots of the average staining scores of *ITGA6* expression in the miR-143-3p-high or miR-143-3p-low tissues (**P < 0.01, *n* = 25 for miR-143-3p-low group; *n* = 24 for miR-143-3p high group). (**e**) Kaplan−Meier overall survival curve of GBC patients based on ITGA6 expression (*P* < 0.05, *n* = 49)

To further evaluate the correlation between *ITGA6* and miR-143-3p in GBC tissues, we examined the levels of *ITGA6* mRNA in the same 49 pairs of GBC tissues and their corresponding NATs. The qRT-PCR results showed that *ITGA6* expression was significantly higher in the GBC tumour tissues than in the corresponding NATs (Fig. 6a and Supplementary Figure S2b). Furthermore, the correlation between miR-143-3p and *ITGA6* in the GBC tissues was evaluated using the Spearman correlation analysis. The Spearman correlation analysis clearly showed a negative correlation between *ITGA6* and miR-143-3p expression in the GBC tissues (Fig. 6b). To further evaluate the negative correlation between *ITGA6* and miR-143-3p, immunohistochemical staining of the tissues with high and low levels of miR-143-3p was performed, and the results showed that the tissues with high miR-143-3p expression had weaker *ITGA6* staining than the tissues with low miR-143-3p expression (Fig. 6c). Additionally, the average staining score for *ITGA6* expression was higher in the miR-143-3p-low group than in the miR-143-3p-high group (**P < 0.01; Fig. 6d). Moreover, the Kaplan−Meier analysis revealed that higher *ITGA6* expression had a short overall survival trend (*P* = 0.0176; Fig. 6e).

### *ITGA6* overexpression rescues the effects of miR-143-3p on GBC cell proliferation and angiogenesis

**Fig. 7:**
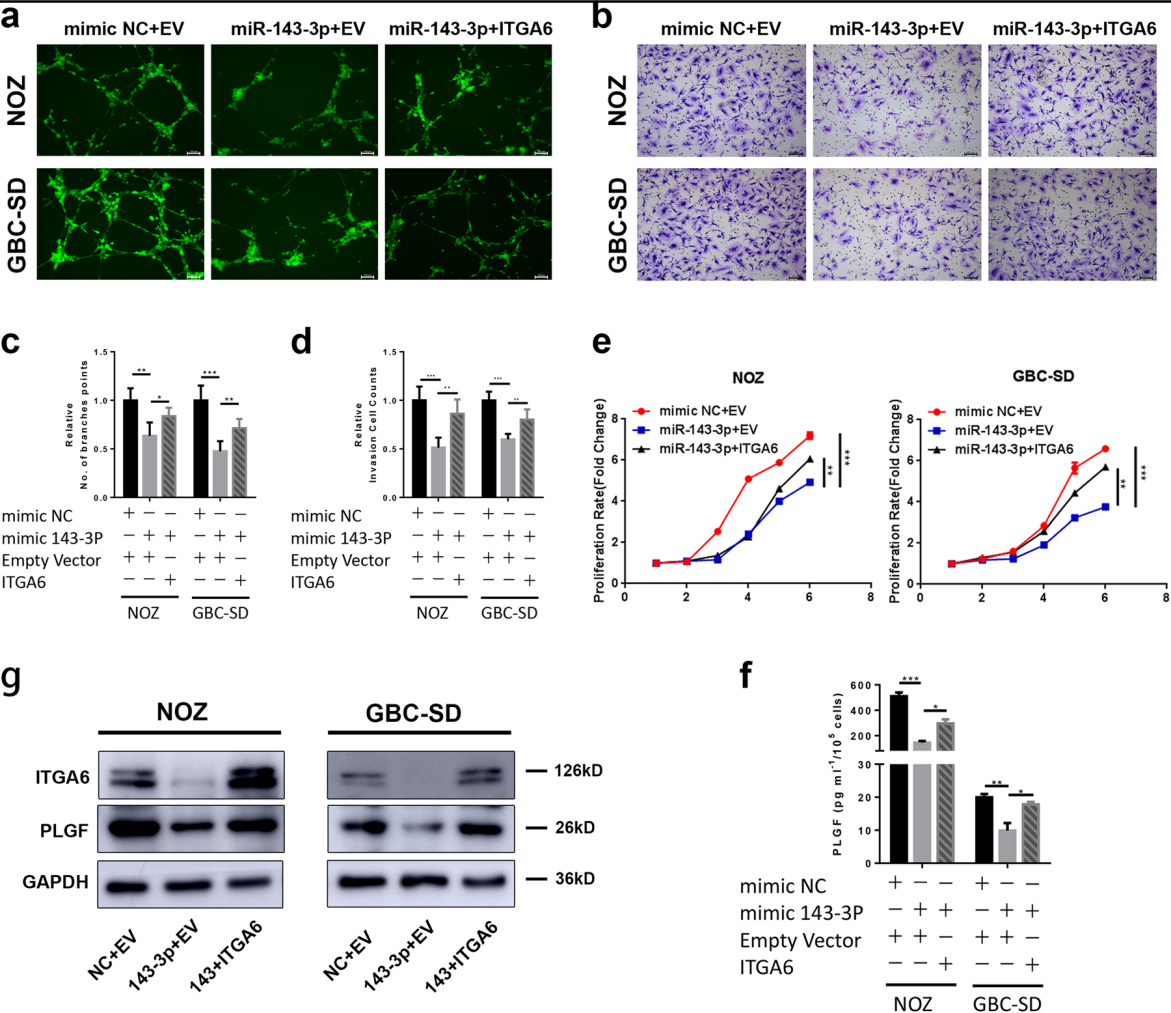
Overexpression of *ITGA6* attenuates the inhibitory effects of miR-143-3p on GBC cells. **a**, **c** Endothelial tube formation was estimated following incubation of HMVECs with conditioned medium from GBC cells transfected with mimic NC or miR-143-3p mimic and empty vector or *ITGA6*. The number of branches was quantified (*P < 0.05, **P < 0.01, ***P < 0.001; Student’s *t* test). Scale bar, 100 µm. **b**, **d** Invasion of HMVECs through the Matrigel chambers after incubation with conditioned medium from GBC cells co-transfected with mimic NC or miR-143-3p mimic and empty vector or *ITGA6* for 48−72 h. Scale bar, 100 µm. The number of invading cells was determined and is depicted in the bar graph (**P < 0.01, ***P < 0.01; Student’s *t* test). **e** The growth rates of the GBC cells that were co-transfected with mimic NC or miR-143-3p mimics and empty vectors or *ITGA6* were determined with CCK-8 proliferation assays (**P < 0.01, ***P < 0.001). **f** *PLGF* in the supernatants from NOZ and GBC-SD cells that were co-transfected with mimic NC or miR-143-3p mimic and empty vector or *ITGA6* was quantified by ELISA (*n* = 3; *P < 0.05, ***P < 0.001; Student’s *t*test). **g** Western blot for the *ITGA6* and *PLGF* proteins in GBC cells that were co-transfected with mimic NC or miR-143-3p mimic and empty vector or *ITGA6*

Rescue experiments were performed to further assess whether the effects of miR-143-3p on GBC cell angiogenesis and proliferation were indeed mediated by *ITGA6*. NOZ and GBC-SD cells were co-transfected with miR-NC or the miR-143-3p mimics together with an empty vector or *ITGA6* vector. The results showed that overexpression of *ITGA6* partially reversed the phenotype caused by overexpression of miR-143-3p (Fig. 7a−e). Furthermore, to determine whether *ITGA6* regulated the expression of *PLGF*, western blot and ELISA were performed to assess *PLGF* levels in the co-transfected cells. The results showed that the levels of *PLGF* increased in the GBC cells transfected with miR-143-3p and *ITGA6* compared with the GBC cells co-transfected with miR-143-3p and empty vector (Fig. 7g, f). Thus, *ITGA6* overexpression can reverse downregulation of *PLGF* by miR-143-3p.

### miR-143-3p downregulates *PLGF* expression through the *ITGA6/PI3K/AKT /STAT3* pathways

**Fig. 8:**
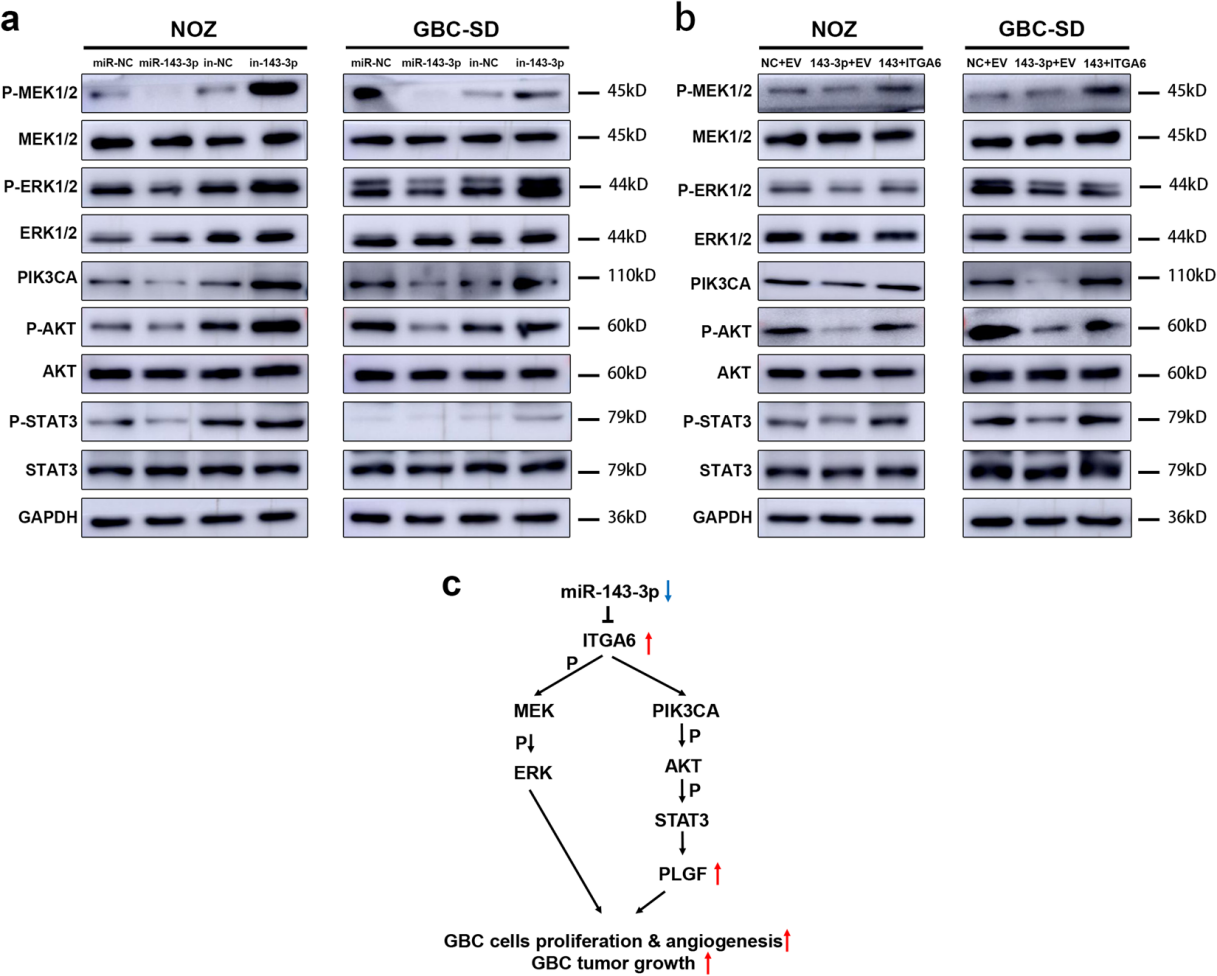
miR-143-3p downregulates *PLGF* expression through the *ITGA6/PI3K/AKT /STAT3* pathways. **a** Western blot analysis of relevant proteins in the *MAPK/ERK* and *PI3K/AKT* pathways in GBC cells that were transfected with mimic (inhibitor) NC or miR-143-3p mimics (inhibitors). **b** Western blot analysis of relevant proteins in the *MAPK/ERK* and *PI3K/AKT* pathways in GBC cells that were co-transfected with mimic NC or miR-143-3p mimic and empty vector or *ITGA6*. **c** Working model of the miR-143-3p regulatory axis in GBC

*ITGA6* activates multiple signal transduction cascades, including *PI3K/AKT* and *MAPK/ERK*^20^. To investigate the molecular mechanism underlying the angiogenesis controlled by miR-143-3p/*ITGA6*, we examined the expression levels of relevant proteins in the *MAPK/ERK* and *PI3K/AKT* pathways. The expression levels of *p-ERK1/2*,* p-MEK1/2*, *PIK3CA*, *p-AKT* and *p-STAT3* were decreased when miR-143-3p was overexpressed (Fig. 8a left). Conversely, the expression levels of *p-ERK1/2*, *p-MEK1/2*, *PIK3CA*, *p-AKT* and *p-STAT3* were increased when miR-143-3p was silenced (Fig. 8a right). These changes were partially recovered by overexpression of *ITGA6*, indicating that miR-143-3p/*ITGA6* functions as a key mediator of angiogenesis via the *MAPK/ERK*and *PI3K/AKT /STAT3* signalling pathways. Moreover, *STAT3* can bind to the promoter of* PLGF* to enhance its expression^21^. We decreased *STAT3* expression by transfecting small interfering RNA (siRNA) against *STAT3*. After siRNA transfection, the expression of *PLGF* was significantly downregulated, which was consistent with the conclusion that *STAT3* enhanced *PLGF* expression (Supplementary Figure S3a-b). Therefore, these findings suggest that miR-143-3p downregulates the expression of *PLGF* through the *ITGA6/PI3K/AKT* pathways (Fig. 8c).

## Discussion

Accumulative evidence indicates that miRNAs serve as oncogenes or tumour suppressors that act as pivotal players in tumourigenesis and tumour progression. Additionally, deregulation of miRNAs has been observed in various cancers, including gastric cancer, liver cancer, pancreatic cancer and colorectal cancer^22–25^. Numerous studies have examined the expression profiles of miRNAs to explore the mechanism of miRNA actions in tumourigenesis and tumour progression. Moreover, new studies have established the potential usefulness of miRNAs as therapeutic molecules against cancer^25^. According to our miRNA profiling results^1^, we found that miR-143-3p was significantly downregulated. We further confirmed this observation in 49 pairs of GBC tissues. Our results further indicated that miR-143-3p inactivated the *MAPK/ERK* and *PI3K/AKT/STAT3* pathways in GBC cells by directly targeting *ITGA6*, resulting in a decrease in *PLGF* and the development of GBC angiogenesis and tumour growth. Therefore, our results reveal a leading mechanism by which miR-143-3p suppresses angiogenesis in GBC.

miR-143-3p acts as a tumour suppressor in various cancers, including lung cancer, colorectal cancer, prostate cancer and hepatocellular carcinoma^26–29^. Low expression of miR-143-3p is associated with a poor clinical outcome. Importantly, increasing evidence has shown the potential usefulness of miRNAs as therapeutic molecules against cancer. Circulating levels of miRNAs are also quite stable and reproducible in the body fluids of many cancer patients. Given the critical roles of miRNAs in tumour development and in physiological regulation overall^30^, further characterizations and screenings of therapeutic targets for GBC hold great promise. However, the precise molecular mechanisms by which miR-143-3p influences various physiological processes of GBC remain unknown. To date, biological functions for miR-143-3p have been reported mainly in areas of proliferation and metastasis, while the impact of miR-143-3p on angiogenesis is not well understood. Therefore, we focused on angiogenesis and performed in vivo and in vitro angiogenesis assays to explore the effects of miR-143-3p on angiogenesis. Through gain- and loss-of-function miR-143-3p assays in GBC cells, we uncovered that miR-143-3p could efficiently weaken in vitro invasion and capillary tube formation of HMVECs and suppress tumour growth and angiogenesis in nude mice. Our results indicate that miR-143-3p is a novel antiangiogenesis miRNA. Angiogenesis has a pivotal function in tumour growth and progression and is responsible for the rapid recurrences of tumours and poor prognoses for patients. Numerous factors are involved in angiogenesis. Among them, *VEGF* plays an important role in various cancers. To determine the target of miR-143-3p that was relevant to inhibition of angiogenesis, an angiogenesis antibody array was performed, and the results indicated that several pro-angiogenesis cytokines were downregulated by miR-143-3p, including *CXCL5, uPAR, VEGFR2, VEGFR3, VEGFA, MMP-9, ANGPT1, TGF-β* and *PLGF*. Among them, *PLGF* showed a sharp decrease. The *ELISA* and western blot analyses also confirmed these results. *PLGF* is a member of the *VEGF* family. Multiple reports have described the relationship between *PLGF*, angiogenesis and metastasis in cancer. For example, *PLGF* promotes metastasis of non-small-cell lung cancer through *MMP9*^31^. To determine whether miR-143-3p directly bound to *PLGF*, we compared the miR-143-3p sequence with the *PLGF* 3′UTR and found no binding site. We presumed that miR-143-3p decreased the expression of *PLGF* by interacting with an upstream regulator of *PLGF*. Therefore, a bioinformatics prediction system (TargetScan, PicTar and miRDB) was used to predict the target of miR-143-3p. According to the results, *ITGA6* was a direct target of miR-143-3p and was associated with angiogenesis. Therefore, we hypothesized that miR-143-3p decreased the expression of *PLGF* by interacting with *ITGA6*. We confirmed this hypothesis with dual-luciferase assays and rescue assays, with follow-up qRT-PCR and western blot assays to further validate the results.

*ITGA6* (integrin α6), also known as *CD49f*, is a member of the integrin family that plays a vital role in the interactions between many cell types and is involved in several biological processes, including cell survival, proliferation and gene transcription^32^. *ITGA6* associates with the integrin β1 chain and integrin β4 to form the *α6β1* or *α6β4* complex. *α6β1* and *α6β4* are important receptors for laminin and are essential for the ECM pathways^20^. Interest in defining the contribution of integrins to transcription has been high. Furthermore, the ability of integrins to regulate translation provides a mechanistic basis for altering cell functions by increasing the expression levels of specific proteins^30^. Recent studies have suggested that *ITGA6* regulates *eIF-4E* activity and *VEGF* translation^33^. Moreover, *ITGA6* is associated with invasion, metastasis and a poor prognosis in human GBC^32,34,35^. In this study, we have shown that *ITGA6* indirectly downregulates *PLGF* via the *PI3K/AKT/STAT3* pathway. Additionally, activation of *AKT* and *STAT3* plays an essential role in tumour development and progression^4^. Moreover, we performed rescue, western blot and ELISA analyses to confirm that miR-143-3p decreased the expression levels of *PLGF* by interacting with *ITGA6*. *ITGA6* overexpression could markedly reverse the inhibitory effects of miR-143-3p on GBC cell proliferation and angiogenesis and on *PLGF* expression. Thus, the antiangiogenic functions of miR-143-3p are indirectly executed by inhibiting its indirect target, *PLGF*.

In summary, our study demonstrated the antiangiogenic effects of miR-143-3p on GBC cells. Furthermore, our study characterized a novel mechanism that underlies the effects of miR-143-3p on GBC cells: the miR-143-3p/*ITGA6/PLGF* axis. Collectively, our results indicate that miR-143-3p and its target *ITGA6* may be effective prognostic indicators. Additionally, more studies of miR-143-3p and *ITGA6* should be conducted so that new approaches for molecular therapeutics that specifically target miR-143-3 and *ITGA6* in GBC patients can be developed.

## Materials and methods

### Tissue samples

Human GBC samples and the corresponding normal gallbladder tissues were obtained from the Department of General Surgery, Xinhua Hospital (Shanghai, China). Additionally, this study was approved by the Research Ethics committee. Written informed consent was obtained from all participants. Tissue samples were collected during surgery and immediately frozen in liquid nitrogen.

### Cell culture and reagents

The GBC-SD and SGC996 human GBC cell lines that were used in this study were purchased from the cell bank of the type culture collection of the Chinese Academy of Sciences (Shanghai, China). NOZ, OCUG-1 and EHGB-1 cells were obtained from the Health Science Research Bank (Osaka, Japan). HMVECs were provided by Dr. Rong Shao^36^. NOZ, GBC-SD, EHGB-1 and OCUG-1 cells were maintained in high-glucose DMEM (Gibco, USA), and HMVECs were maintained in ECM (ScienCell, USA) supplemented with 10% FBS (Gibco, USA), penicillin G (100 U/ml) and streptomycin (100 g/ml). The SGC996 cells were cultured in Roswell Park Memorial Institute (Gibco, USA) 1640 medium supplemented with 10% FBS, penicillin G (100 U/ml) and streptomycin (100 g/ml). Cells were maintained as monolayer cultures at 37 °C in humidified air with 5% CO_2_ and 95% air. Before the experiments, cell lines were validated by microscopy, growth curve analysis and mycoplasma detection according to the cell line verification test recommendations.

### RNA extraction and quantitative real-time PCR

Total RNA was extracted from the tissue samples or cells using TRIzol reagent (Invitrogen, Carlsbad, CA, USA). Quantitative real-time PCR was performed using SYBR^®^ Green (Takara, Japan) according to the manufacturer’s instructions. The qRT-PCR results were analysed and examined as the relative miRNA or mRNA levels based on cycle threshold (CT) values, which were converted to fold changes. The primer sequences used are listed in Supplementary Table S1.

### Cell transfection

Hsa-miRNA mimics, hsa-miRNA inhibitors and their cognate control RNAs were purchased from Riobio (Guangzhou, China). Lentivirus-miR-143-3p and lentivirus-miR-NC were purchased from Genomeditech (Shanghai, China). Additionally, an *ITGA6* overexpression plasmid was used for the rescue experiments. The miRNA mimics, miRNA inhibitors and plasmids were transfected into NOZ and GBC-SD cells using Lipofectamine 2000 transfection reagent (Invitrogen, USA). Total RNA and protein were collected 48 h after transfection.

### Generation of stable cell lines with overexpression of miR-143-3p

For the construction of cell lines stably expressing miR-143-3p, miR-143-3p and negative control sequence were synthesized and inserted into PGMLV-hU6-MCS-CMV-ZsGreen1-PGK-puromycin-WPRE lentiviral vector. Recombinant lentiviruses expressing miR-143-3p or negative control (Lv-miR-143-3p and Lv-miR-NC, respectively) were produced by Genomeditech (Shanghai, China). NOZ cells were infected with concentrated virus, and the culture medium was replaced after 24 h incubation. Then, cells were treated with 1 μg/ml puromycin for 2 weeks for the selection of stable cell lines. The expression of miR-143-3p in the stable cell lines and xenograft tumour was validated by qRT–PCR analysis.

### Human angiogenesis antibody array

The human angiogenesis antibody array kit was purchased from RayBiotech (AAH-ANG-1000-4, Norcross, GA). A total of 1×10^5^ NOZ cells transfected with mimic NC or miR-143-3p mimics were seeded onto a six-well plate with 1 ml of serum-free medium. Forty-eight hours later, the conditioned medium was collected and used for a human angiogenesis antibody array analysis. The procedure was performed as per the manufacturer’s instructions. Briefly, 2 ml of blocking buffer was pipetted into the angiogenesis antibody membrane and incubated for 30 min at room temperature (RT). After the incubation, the blocking buffer was aspirated. The antibody membrane was washed twice with the array wash buffer. Afterwards, 1 ml of conditioned medium was added into the well, and the antibody membranes were incubated overnight at 4 °C. Two millilitres of the HRP-Streptavidin solution was added onto the membrane and incubated for 2 h at RT. A chemiluminescent substrate ECL kit was used to obtain detailed pictures of the array.

### Enzyme-linked immunosorbent assay

Enzyme-linked immunosorbent assay was used to measure the levels of *PLGF* in the cancer cell supernatants. The human *PLGF* ELISA kit (Boster, Wuhan, China) was used according to the manufacturer’s instructions.

### In vitro tube formation and invasion assay

Ten microliters of ice-cold Matrigel (BD Matrigel^TM^, USA) was added into culture plates (µ-Slide Angiogenesis, ibidi, USA). Cells (2×10^4^/well) in 50 μl of conditioned medium were then seeded onto these plates. After incubation for 4–6 h at 37 °C, Calcein AM (Sigma, USA) was added to the plates. Tube formation was examined using photographs obtained from fluorescence microscopy examinations (Leica, Germany). The number of branch points was determined and analysed in five random fields per replicate. For the invasion assays, HMVECs (2×10^4^/well) in 200 μl of FBS-free DMEM were added into the upper chambers, while the lower chambers were filled with 600 μl of conditioned medium. After 48−72 h of incubation, the filters were removed and fixed with 4% paraformaldehyde for 30 min. The cells located on the bottom sides of the filters were stained with crystal violet for 20 min. The stained cells that had invaded were counted in five randomly chosen fields (×100 magnification) per well.

### Subcutaneous xenograft models

The subcutaneous xenograft model was established using 4- to 6-week-old male BALB/c athymic nude mice. The mice were purchased from the Shanghai Laboratory Animal Center of the Chinese Academy of Sciences (Shanghai, China). The mice were housed under specific pathogen-free conditions and fed a regular autoclaved chow diet with water ad libitum. A total of 1×10^6^ NOZ cells stably expression miR-143-3p or negative control (Lv-miR-143-3p or Lv-miR-NC) were inoculated subcutaneously into the ventral areas of the mice (*n* = 5 per group). The sizes of the tumours were measured every 2 days after inoculation. Additionally, at the endpoint (approximately 3 weeks), the weights of the tumours were measured. The sizes were evaluated using the following formula: tumour volume = (tumour length × (tumour width)^2^) × 0.52. After that, the tumours were fixed and used for in situ hybridization (ISH) and IHC assays. All animal experiments were approved by the Institutional Animal Care and Use Committee of Xinhua Hospital (2013-0106) and conducted humanely.

### **In vivoMatrigel plug assay**

NOZ cells were infected with a negative control lentivirus or a mir-143-3p lentivirus (Lv-miR-NC or Lv-miR-143-3p) and were selected with 0.5 µg/ml of puromycin for 10 days. A total of 0.5 ml of Matrigel (BD Biosciences, USA) containing 20 U of heparin and 1×10^6^ infected NOZ cells was injected subcutaneously into the ventral areas of BALB/c athymic nude mice (*n* = 5 per group). After 7 days, mice were killed, and the plugs were removed. The plugs were photographed, fixed and stained for H&E and *CD31* analyses.

### Luciferase reporter assay

To investigate the binding site of miR-143-3p and its candidate target gene *ITGA6*, dual-luciferase reporter assays were performed using 293T cells with the pmirGLO System (Promega, USA) following the manufacturer’s protocol. Cells were co-transfected with 50 nM miR-143-3p mimic, the inhibitor or the cognate controls and 0.5 μg of pmirGLO-*ITGA6*-3′UTR-WT/pmirGLO-*ITGA6*-3′UTR-MUT vector. After 48 h, cells were collected, and the luciferase activity was analysed according to the manufacturer’s protocol (Promega, USA).

### Immunofluorescence

After transfections with the mimics or inhibitors, GBC cells were seeded onto 24-well plates. Two days later, the cells were washed with PBS and fixed in 4% for 30 min at RT. Afterwards, cells were incubated with blocking buffer (5% BSA, 0.1% Triton X-100) alone for 1 h and with the primary antibodies overnight at 4 °C. The primary antibody was removed, and cells were incubated with the secondary fluorescent antibody (594) for 1 h at RT. After several PBS washes, cells were stained with DAPI and photographed under a fluorescence microscope (Leica, Germany).

### Immunohistochemistry

Immunohistochemistry was performed using the anti-*ITGA6* (Sigma, 1:200), anti-*Ki67* (Proteintech, 1:400), anti-*PLGF* (Abcam, 1:200) and anti-mouse *CD31* (Abcam, 1:400) antibodies. Frozen or paraffin-embedded sections were used for immunohistochemical analysis. For immunohistochemical staining, tissue sections were first incubated with 0.1% trypsin at RT for 10 min followed by incubation with 0.1 μg/ml trypsin inhibitor (Sigma) for 5 min. The sections were then rinsed three times in PBS before blocking with 10% normal goat serum (15 min at RT). The sections were rinsed in PBS and incubated with a primary antibody overnight at 4 °C. Afterwards, the tissue sections were rinsed three times in PBS and incubated with the secondary antibody for 60 min at RT. The sections were then immersed in DAB for 5−10 min and counterstained with 10% Mayer’s haematoxylin. *ITGA6* expression in tissues was evaluated according to the methods described by R Shao et al ^37^.

### In situ hybridization

The triple digoxigenin-labelled antisense locked nucleic acid (LNA)-modified probes for miR-143-3p were synthesized by Boster Biotech (Wuhan, China). ISH was conducted according to the manufacturer’s instruction of microRNA ISH Optimization Kits (Boster, Wuhan, China).

### Statistical analysis

All experiments were repeated three times unless otherwise noted. The data are presented as the mean ± SD. Student’s *t* test was used for single comparisons, and one-way ANOVA was used for multiple comparisons. Kaplan−Meier curves and log-rank tests were performed to compare the survival times among the groups. All data were analysed with GraphPad Prism 5 and SPSS v17.0 software. *P* < 0.05 was considered statistically significant.

## Electronic supplementary material

Supplementary Figure S1

Supplementary Figure S2

Supplementary Figure S3

Supplementary Information

Table S1

## References

[1] Shu Y (2017). MicroRNA-29c-5p suppresses gallbladder carcinoma progression by directly targeting CPEB4 and inhibiting the MAPK pathway. Cell Death Differ..

[2] Gourgiotis S (2008). Gallbladder cancer. Am. J. Surg..

[3] Shu Y (2015). SPOCK1 as a potential cancer prognostic marker promotes the proliferation and metastasis of gallbladder cancer cells by activating the PI3K/AKT pathway. Mol. Cancer.

[4] Zhang Y (2016). A novel PI3K/AKT signaling axis mediates Nectin-4-induced gallbladder cancer cell proliferation, metastasis and tumor growth. Cancer Lett..

[5] Hundal R, Shaffer E (2014). Gallbladder cancer: epidemiology and outcome. Clin. Epidemiol..

[6] Li M (2014). Whole-exome and targeted gene sequencing of gallbladder carcinoma identifies recurrent mutations in the ErbB pathway. Nat. Genet..

[7] Li Z (2016). LASP-1 induces proliferation, metastasis and cell cycle arrest at the G2/M phase in gallbladder cancer by down-regulating S100P via the PI3K/AKT pathway. Cancer Lett..

[8] Wu S (2016). A miR-192-EGR1-HOXB9 regulatory network controls the angiogenic switch in cancer. Nat. Commun..

[9] De Palma M, Biziato D, Petrova T (2017). Microenvironmental regulation of tumour angiogenesis. Nat. Rev. Cancer.

[10] Hicklin D, Ellis L (2005). Role of the vascular endothelial growth factor pathway in tumor growth and angiogenesis. J. Clin. Oncol..

[11] De Falco S (2012). The discovery of placenta growth factor and its biological activity. Exp. Mol. Med..

[12] Lee J (2017). Safety and clinical activity of the programmed death-ligand 1 inhibitor durvalumab in combination with poly (ADP-ribose) polymerase inhibitor olaparib or vascular endothelial growth factor receptor 1-3 inhibitor cediranib in women’s cancers: a dose-escalation, phase I study. J. Clin. Oncol..

[13] Faivre S (2017). Sunitinib in pancreatic neuroendocrine tumors: updated progression-free survival and final overall survival from a phase III randomized study. Ann. Oncol..

[14] Hatem R (2016). Vandetanib as a potential new treatment for estrogen receptor-negative breast cancers. Int. J. Cancer.

[15] Liang Y (2017). The EGFR/miR-338-3p/EYA2 axis controls breast tumor growth and lung metastasis. Cell Death Dis..

[16] Liu K (2017). SOX2 regulates multiple malignant processes of breast cancer development through the SOX2/miR-181a-5p, miR-30e-5p/TUSC3 axis. Mol. Cancer.

[17] Liu Y (2017). miR-19a promotes colorectal cancer proliferation and migration by targeting TIA1. Mol. Cancer.

[18] Li G, Pu Y (2015). MicroRNA signatures in total peripheral blood of gallbladder cancer patients. Tumour Biol..

[19] Letelier P (2014). miR-1 and miR-145 act as tumor suppressor microRNAs in gallbladder cancer. Int. J. Clin. Exp. Pathol..

[20] Stewart R, O’Connor K (2015). Clinical significance of the integrin α6β4 in human malignancies. Lab Invest..

[21] Tu H (2013). Enhancement of placenta growth factor expression by oncostatin M in human rheumatoid arthritis synovial fibroblasts. J. Cell Physiol..

[22] Han T (2015). MicroRNA-29c mediates initiation of gastric carcinogenesis by directly targeting ITGB1. Gut.

[23] Chang R (2014). MicroRNA-331-3p promotes proliferation and metastasis of hepatocellular carcinoma by targeting PH domain and leucine-rich repeat protein phosphatase. Hepatology.

[24] Imamura T (2017). Depleted tumor suppressor miR-107 in plasma relates to tumor progression and is a novel therapeutic target in pancreatic cancer. Sci. Rep..

[25] Xiao J (2017). Therapeutic inhibition of miR-4260 suppresses colorectal cancer via targeting MCC and SMAD4. Theranostics.

[26] Liu X, Gong J, Xu B (2015). miR-143 down-regulates TLR2 expression in hepatoma cells and inhibits hepatoma cell proliferation and invasion. Int. J. Clin. Exp. Pathol..

[27] Su J (2014). MiR-143 and MiR-145 regulate IGF1R to suppress cell proliferation in colorectal cancer. PLoS ONE.

[28] Wei J (2015). miR-143 inhibits cell proliferation by targeting autophagy-related 2B in non-small cell lung cancer H1299 cells. Mol. Med. Rep..

[29] Wu D (2013). MicroRNA-143 inhibits cell migration and invasion by targeting matrix metalloproteinase 13 in prostate cancer. Mol. Med. Rep..

[30] Li L (2016). Serum miRNAs as predictive and preventive biomarker for pre-clinical hepatocellular carcinoma. Cancer Lett..

[31] Zhang W (2015). Placental growth factor promotes metastases of non-small cell lung cancer through MMP9. Cell Physiol. Biochem..

[32] Lowell C, Mayadas T (2012). Overview: studying integrins in vivo. Methods Mol. Biol..

[33] Chung J (2002). Integrin (alpha 6 beta 4) regulation of eIF-4E activity and VEGF translation: a survival mechanism for carcinoma cells. J. Cell Biol..

[34] Zhang D (2016). Overexpression of Thy1 and ITGA6 is associated with invasion, metastasis and poor prognosis in human gallbladder carcinoma. Oncol. Lett..

[35] Pabla R (1999). Integrin-dependent control of translation: engagement of integrin alphaIIbbeta3 regulates synthesis of proteins in activated human platelets. J. Cell Biol..

[36] Shao R, Guo X (2004). Human microvascular endothelial cells immortalized with human telomerase catalytic protein: a model for the study of in vitro angiogenesis. Biochem. Biophys. Res. Commun..

[37] Shao R (2009). YKL-40, a secreted glycoprotein, promotes tumor angiogenesis. Oncogene.

