# Supplementary figures and images for: miR-143-3p targeting of ITGA6 suppresses tumour growth and angiogenesis by downregulating PLGF expression via the PI3K/AKT pathway in gallbladder carcinoma
Source: Cell Death Dis. 2018 Feb 7;9(2):182. doi: 10.1038/s41419-017-0258-2 (PMC5833358; doi:10.1038/s41419-017-0258-2)

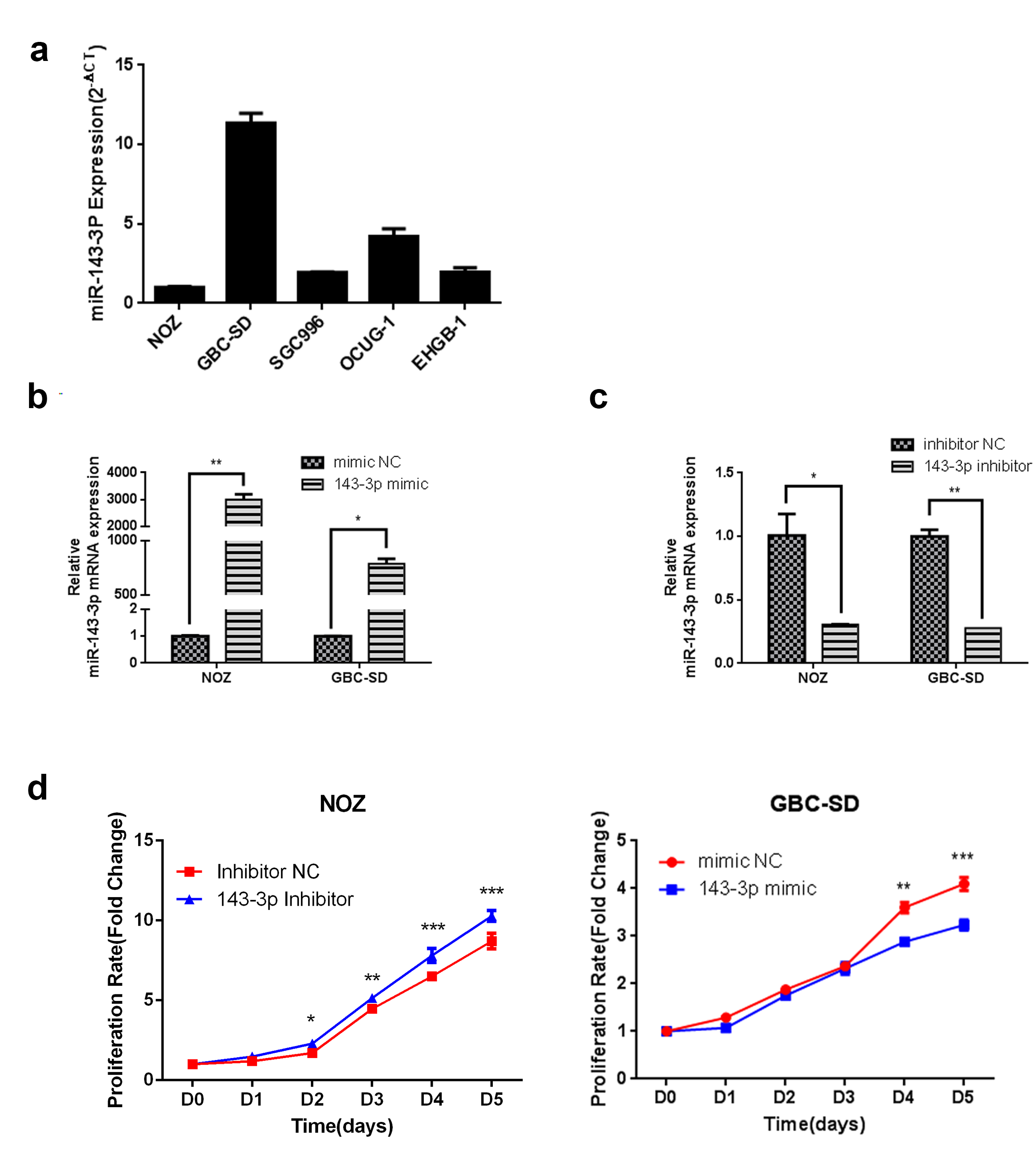

Supplement: Supplementary file 1 — Supplementary Figure S1 [file 41419_2017_258_MOESM1_ESM.tif]

a

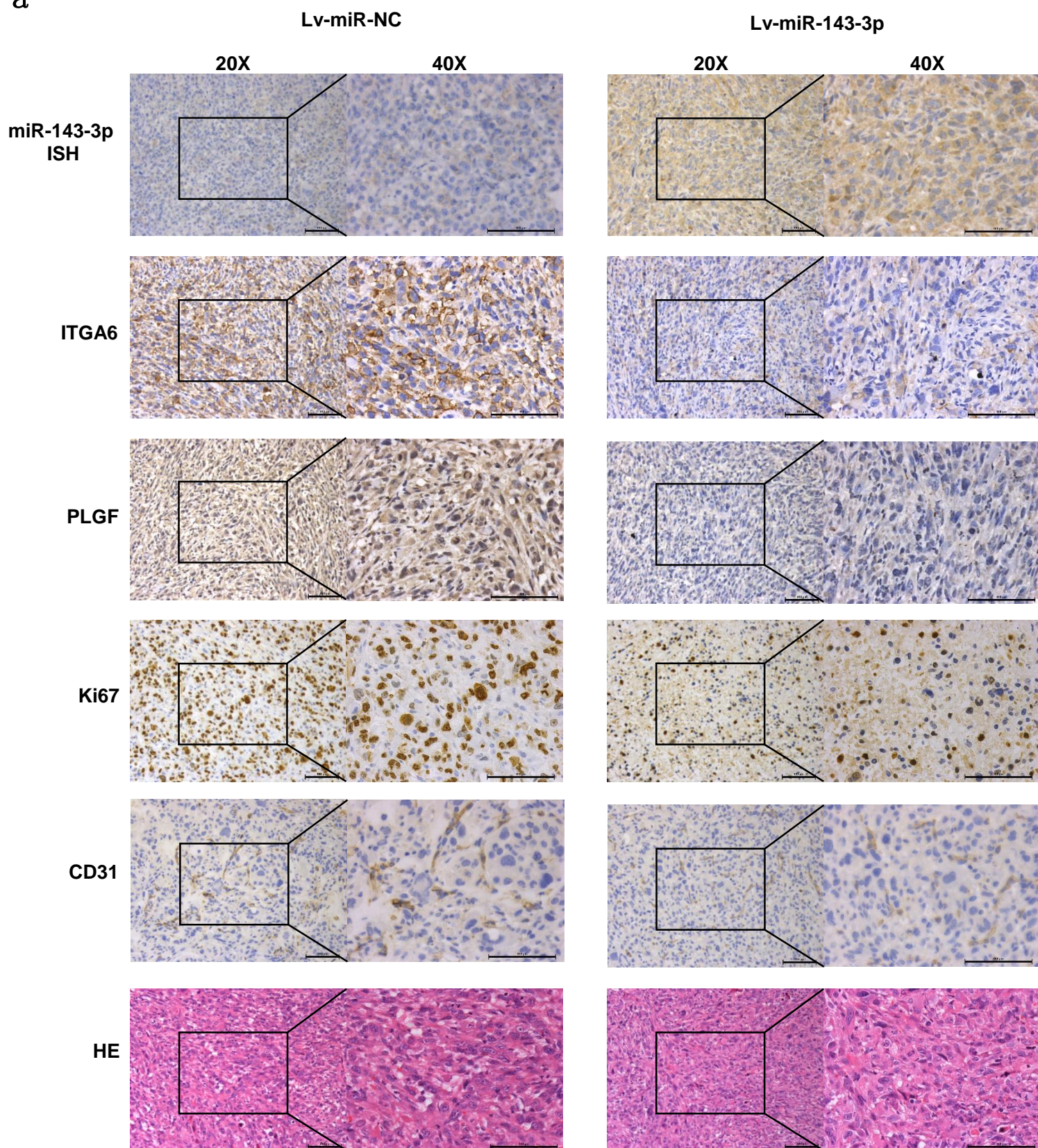

b

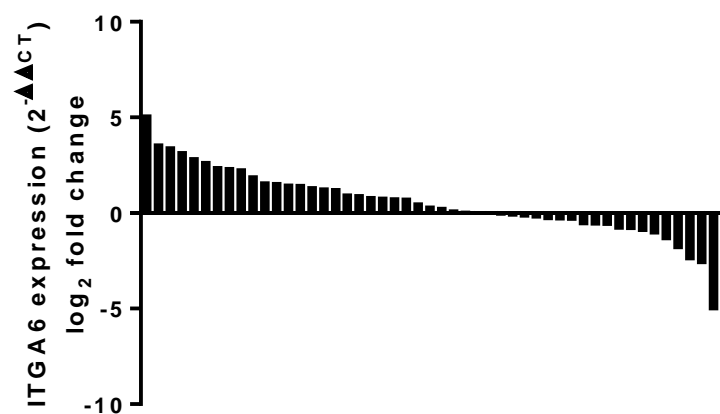

c

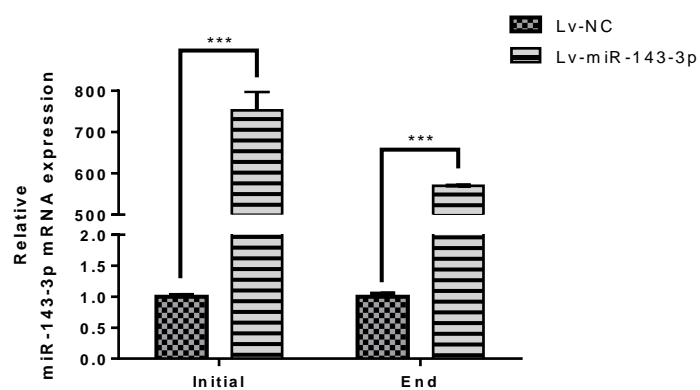

Supplement: Supplementary file 2 — Supplementary Figure S2 [file 41419_2017_258_MOESM2_ESM.pdf]

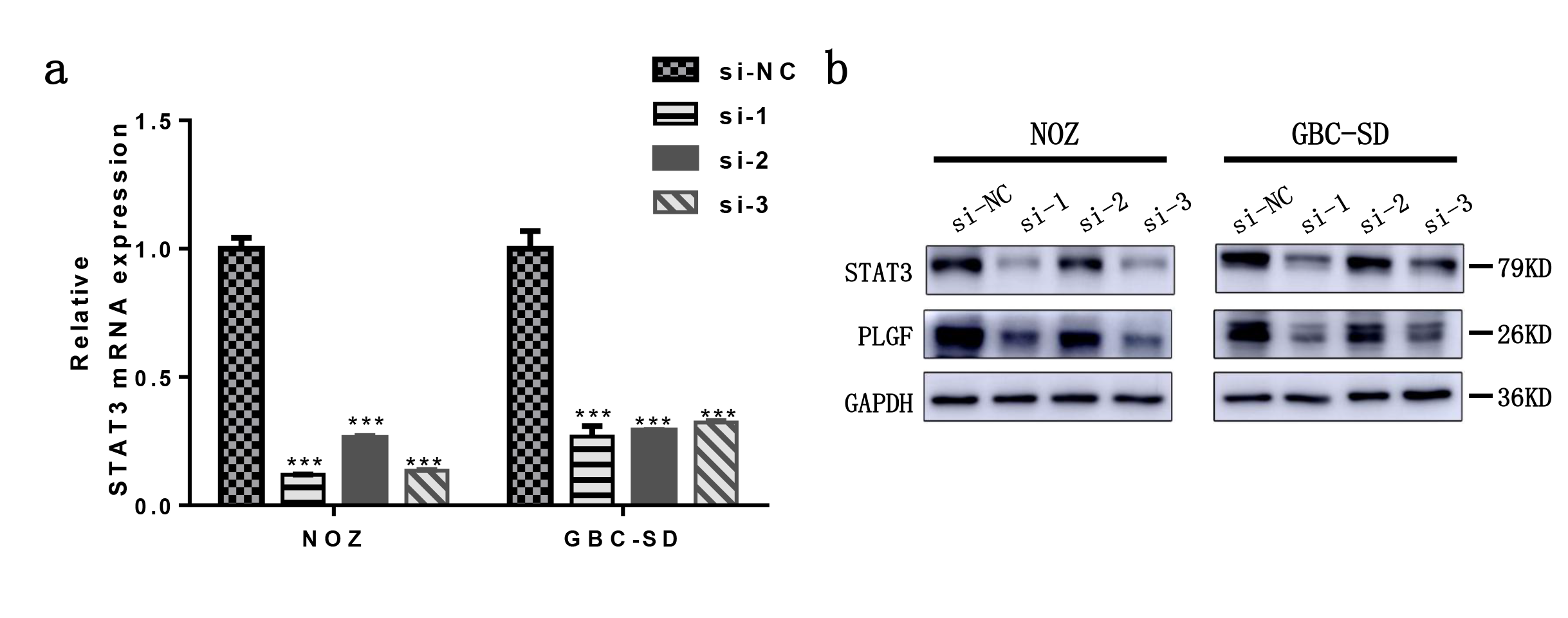

Supplement: Supplementary file 3 — Supplementary Figure S3 [file 41419_2017_258_MOESM3_ESM.tif]
