# Supplementary material for: miR-143-3p targeting of ITGA6 suppresses tumour growth and angiogenesis by downregulating PLGF expression via the PI3K/AKT pathway in gallbladder carcinoma
Source: Cell Death Dis. 2018 Feb 7;9(2):182. doi: 10.1038/s41419-017-0258-2 (PMC5833358; doi:10.1038/s41419-017-0258-2)
Supplement: Supplementary file 4 — Supplementary Information [file 41419_2017_258_MOESM4_ESM.docx]

**Supplementary Figure Legends**

**Supplementary Figure S1.** (**a**) miR-143-3p expression in 5 human GBC cell lines. (**b**) The miR-143-3p mimic and mimic NC were transfected into GBC cells, and the transfection efficiency was determined by qRT-PCR (**P*<0.05, ***P*<0.01). (**c**) The growth rates over 5 days of NOZ cells transfected with the miR-143 inhibitor or inhibitor NC and GBC-SD cells transfected with the miR-143-3p mimic or mimic NC were determined with CCK-8 proliferation assays (***P*<0.01, ****P*<0.001).

**Supplementary Figure S2.** (**a**) ISH of miR-143-3p and IHC for Ki67, ITGA6, PLGF, CD31 and H&E in GBC xenograft tumours. (**b**) ITGA6 expression in GBC tissues and NATs from same 49 patients (qRT-PCR; GAPDH as the internal control; Wilcoxon matched-pairs test). (**c**) Quantitative RT-PCR analysis of miR-143-3p levels in stable re-expression of miR-143-3p NOZ cells (the initial cells and the end xenograft tumours, ****P*<0.001).

**Supplementary Figure S3.** (**a**) Quantitative RT-PCR analysis of STAT3 levels in NOZ and GBC cells transfected with STAT3 siRNA (si-1, si-2 and si-3) or si-NC (****P*<0.001). (**b**) Western blot analysis of STAT3 and PLGF in NOZ and GBC-SD cells after transfection with STAT3 siRNA (si-1, si-2 and si-3) or si-NC (****P*<0.001).
