# Supplementary material for: miR-143-3p targeting of ITGA6 suppresses tumour growth and angiogenesis by downregulating PLGF expression via the PI3K/AKT pathway in gallbladder carcinoma
Source: Cell Death Dis. 2018 Feb 7;9(2):182. doi: 10.1038/s41419-017-0258-2 (PMC5833358; doi:10.1038/s41419-017-0258-2)
Supplement: Supplementary file 5 — Table S1 [file 41419_2017_258_MOESM5_ESM.docx]

**Table S1. Primer sequences**

| Target ID |  | Sequence |
| --- | --- | --- |
| Hsa-miR-143-3p | Forward | GCGCTGAGATGAAGCACTG |
| ITGA6 | Forward | GAGCTTTTGTGATGGGCGATT |
|  | Reverse | CTCTCCACCAACTTCATAAGGC |
| PLGF | Forward | TGCTGCGGCGATGAGAATC |
|  | Reverse | GTCTCCTCCTTTCCGGCTT |
| VEGFA | Forward | CAACATCACCATGCAGATTATGC |
|  | Reverse | GCTTTCGTTTTTGCCCCTTTC |
| GAPDH | Forward | GTCGCCAGCCGAGCCACATC |
|  | Reverse | CCAGGCGCCCAATACGACCA |
| U6 | Forward | CTCGCTTCGGCAGCACA |
|  | Reverse | AACGCTTCACGAATTTGCGT |
